# Pre-Sowing Treatments Improve Germinability of South Texas Native Plant Seeds

**DOI:** 10.3390/plants10112545

**Published:** 2021-11-22

**Authors:** Kaitlynn Lavallee, Pushpa Gautam Soti, Hansapani Rodrigo, Rupesh Kariyat, Alexis Racelis

**Affiliations:** 1 School of Earth, Environment and Marine Sciences, University of Texas—Rio Grande Valley, Edinburg, TX 78539, USA; katie@texaslocalfood.org; 2 Department of Biology, University of Texas—Rio Grande Valley, Edinburg, TX 78539, USA; 3 School of Mathematical and Statistical Sciences, University of Texas—Rio Grande Valley, Edinburg, TX 78539, USA; hansapani.rodrigo@utrgv.edu

**Keywords:** seed dormancy, seed treatment, native plants, habitat restoration

## Abstract

The incorporation of native plant species is central to restoration efforts, but this is often limited by both the availability of seeds and the relatively low viability and germination rates of commercially available seeds. Although pre-sowing treatments are commonly used to improve germination rates of seeds, the efficacy of these treatments is found to vary across species. In this study, we tested how four pre-sow treatments (physical scarification, acid scarification, cold stratification, and aerated hydropriming) affected the viability and seed germination rates of 12 commercially available plant species native to south Texas and commonly used in restoration efforts. Our results show that the viability of the seeds have a wide range, from 78% to 1.25%. Similarly, the total germination rate ranged from 62% to 0%. We found that pre-sowing treatments accelerated the germination rate in 9 of 12 plant species tested, but the effect varied by treatment. Collectively, our results identify various methods to achieve the best germination rates for native plants of south Texas, to help improve restoration efforts across the region.

## 1. Introduction

Native plants are a key element for habitat restoration and landscaping projects [1] as they are well adapted to local environments, and often have fewer resource requirements, lower maintenance needs [2], and contribute to biodiversity and ecosystem stability [3]. These plants also assist in soil stabilization, erosion control, and reduce chemical runoff from entering waterways [4,5]. Furthermore, native plants provide tremendous ecosystem services when incorporated into agroecosystems. For example, native plants implemented in horticultural systems host lower densities of pests [6] and, compared to non-native plants, they also attract higher numbers of natural enemies [7,8], showing promise for incorporating into integrated pest management strategies. However, the use of native plants in landscaping and habitat restoration is often impeded by the lack of seed availability and reduced viability.

With the increase in demand of native plants in habitat restoration projects and landscaping, commercial growers have started to respond to this demand. Seeds produced in commercial farms face significantly different environmental variables compared to that in nature where low seed viability and seed dormancy may improve plant fitness as adaptations for seed herbivory and successful establishment [9,10,11]. Furthermore, seed management and storage conditions vary among commercial operations and can also influence viability and result in lower germination rates [12]. Seed germination is influenced by a variety of environmental factors including soil moisture, fire, temperature, pH, seed burial depth, light, and soil tillage [13,14]. These environmental factors can therefore be simulated as pre-sowing treatments to improve germination rates [15,16]. Since the success of restoration projects is often constrained by costs, pre-sow treatments that increase germination rates improve restoration efforts by reducing seed loss [17].

Pre-sowing techniques are often designed to mimic environmental events that break seed dormancy. For example, sand scarification mimics seed damage from ungulate trampling and digging mammals [18]. Acid scarification emulates endozoochory, simulating seeds traveling through digestive tracts of granivorous birds and mammals [19,20]. On the other hand, seeds from some species tend to stay dormant until exposed to a certain amount of time in either cold or warm temperatures or damp conditions. Cold stratification, or the purposeful manipulation of temperature regimes, imitate natural winter conditions [21,22], while aerated hydropriming recreates the natural occurrence of heavy and seasonal precipitation. In this study, we focus on these four pre-sowing treatments as they mimic natural events common in the semi-arid region of south Texas, USA, the focus area of this study.

## 2. Results

**Figure 1:**
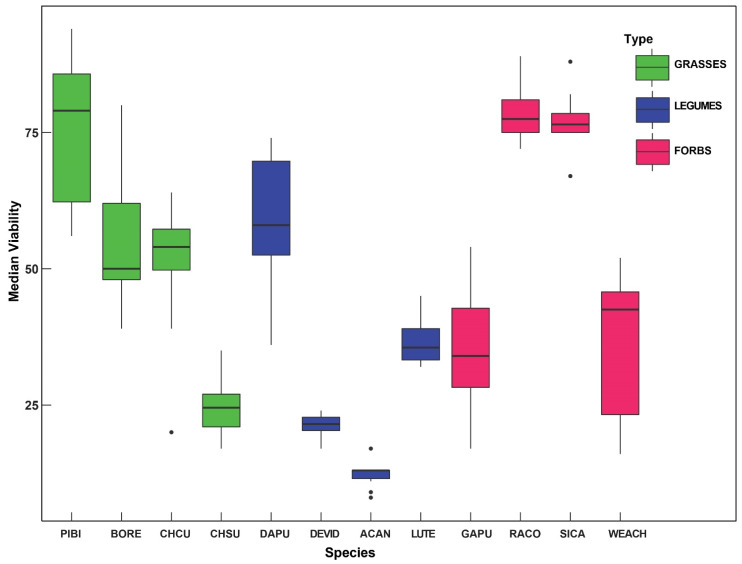
Box plot representing the viability percentage of each species with significant differences denoted between all the species. Species are subcategorized by plant type, *P. bicolor* (PIBI)*,* *B. repens* (BORE), *C. cucullata* (CHUC)*, C. subdolistachya* (CHSU), *D. purpurea* (DAPU), *D. virgatus* (DEVID), *A.* *angustissima* (ACAN), *L. texensis* (LUTE), *G. puchella* (GAPU), *R. columnifera* (RACO), *S. calva* (SICA), and *W. acapulcensis* (WEACH).

Plant species used in this study varied in both seed viability and germination rates (Table 1, Figure 1). *Ratibidia columnifera* had the highest viability (78.7%) as well as germination rate (62%) in contrast to *Acaciella angustissima* which had lowest viability (12.3%) and *Gaillardiapulchella and W edelia acapulcensis* with 0% germination. Similarly, *Desmanthus virgatus*, *B. repens*, *Chloris subdolistachya*, *Dalea purpurea*, and *W. acapulcensis* had <10% median germination.

As mentioned earlier, species with <10% overall germination (see Table 1) were not included in further analysis, with exception to include *D. virgatus* which had a total median germination of 8%. *D. virgatus* was included in further analysis as it had significantly higher germination rate under sand scarification treatment compared to control (p<0.001), justifying its selection for analysis.

**Figure 2:**
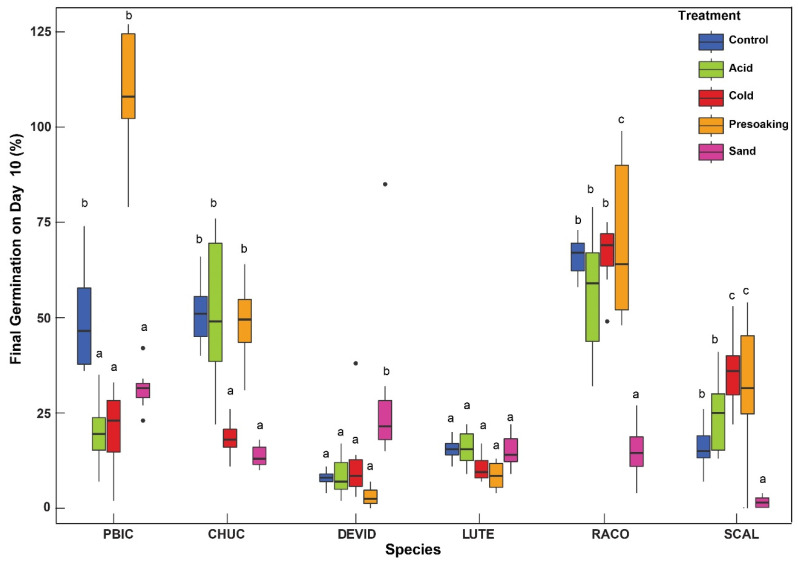
A representation of the final germination (FG) on day 10 for select species of germination >10%. Species meeting the standards included in this analysis are *P. bicolor* (PBIC)*,* *C. cucullata* (CHUC)*, D. virgatus* (DEVID, *L. texensis* (LUTE), *R. columnifera* (RACO), and *S. calva* (SICA). Significant differences in the final germination within each species were evaluated using multiple comparisons-adjusted Tukey’s HSD test.

We found significant differences between the germination of the 12 study species (Figure 2) (F(11, 227)=91.82, p<0.001) with a large effect size (η^2^ = 0.82; 95% CI: 0.78, 0.84). Bonferroni-adjusted pairwise comparisons with *t*-test (59/66 different species combinations) revealed significant differences in total median germination. For instance, significance differences in the median germination were observed among, flowering forbs of *R. columnifera* and *G. pulchella* (p<0.001) as well as *S. calva* and *G. pulchella* (p<0.001). Grass species germination *C. subdolyistachya* was significantly different from *P. bicolor* (p<0.001) and *C. cuculatta* (p<0.001). Significant differences were also seen in legumes species *D. virgatus* and *D. purpurea* (p<0.001) and *L. texensis* and *D. purpurea* (p<0.001).

The interaction between treatments and individual species for the top contenders on the total germination were analyzed using a Two-way ANOVA with unbalanced design (Type-III sums of squares has been used). Significant differences in total germination were observed among different species (F(11, 550)=91.46, p<0.001), different treatments (F(4, 550)=2.80,p<0.05), and their interaction (F(44, 550)=37.03,p<0.001) with large effect sizes. The corresponding partial eta squared for species, treatments, and their interaction were 0.86 (95% CI: 0.80,0.87), 0.34(95% CI: 0.28,0.39), and 0.75 (95% CI: 0.71,0.77), respectively, indicating large effect sizes.

### Time to Germination

**Figure 3:**
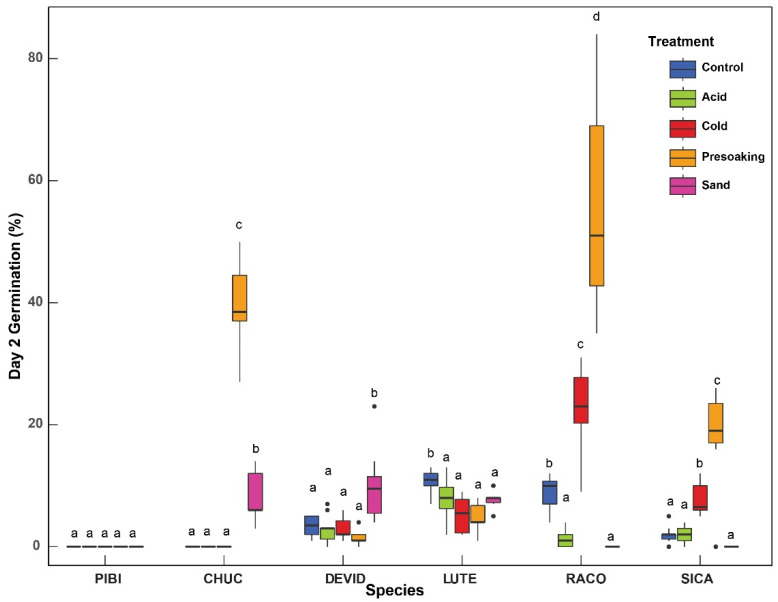
A representation of differences in germination on day 2, between treatments within each selected Species. Significant differences on day 2 germination within each species were evaluated using multiple comparisons-adjusted Tukey’s HSD test. *P. bicolor* (PIBI)*, C. cucullata* (CHUC), *D. virgatus* (DEVID, *L. texensis* (LUTE), *R. columnifera* (RACO), *S. calva* (SICA). Significant determinants (*p* < 0.05) are denoted within species with letter characters.

As per two-way ANOVA model, significant differences in germination on the second day were also observed among different species (Figure 3) (F(11, 550)=18.05, p<0.001), different treatments (F(4, 550)=4.51,p<0.01), and their interaction (F(44, 550)=67.96,p<0.001) with large effect sizes, 0.74 (95% CI: 0.70,0.76), 0.55 (95% CI: 0.50,0.59), and 0.84 (95% CI: 0.82,0.86), respectively. We noticed that the study species in general, germinate sooner with treatment than without. In all species, except *P. bicolor* and *L. texensis*, a seed treatment accelerated germination early on based on day two results (Figure 3). Of the top candidates we explored further, 50% of the study species had the highest germination output with the SP treatment. In particular, the SP treatment for *R. columnifera* was significantly (M=55.5, SD=16.61) productive early on with 5x the germination compared to the control (M=9.1, SD=2.77; p<0.001). By the second day, the SP treatment of *C. cucullata* stimulated germination by ~40%, (M=39.9, SD=7.05) compared to the control with no germination (p<0.001). *S. calva* also displayed significantly improved germination (M=18.4, SD=7.34) with the treatment, resulting in nearly 90% of its total germinal output by the second day compared to control (M=2, SD=1.33;p<0.001). The sand scarification also resulted in significantly different germination (M=2.62, SD=4.37) compared to other treatments—acid (M=1.23, SD=2.49;p<0.01), cold (M=3.57, SD=6.55;p<0.05) and presoaking (M=10.7, SD=18.4;p<0.001).

The best germination results throughout the entire experiment (within all treatments and between all species) were found in *P. bicolor*. The SP on *P. bicolor* did significantly well with 100% germination overall (p<0.001) compared to control. There were no significant differences among control vs. AS (p=1.000), and control vs. AH (p=1.000) treatments in the *C. cucullata* grass species. The SP treatment has a high standard error (SE) of 34.4 in the total germination.

Overall, *R. columnifera* had the highest germination rate of all species (Table 1). All treatments, except sand scarification, produced mean germination results >50%. There was no significant difference between in final germinations among presoak and control (p=0.100), and cold vs. presoak (p=1.000).

When analyzing the *S. calva*, there is no significant difference between CS and AS (p<0.001) or between AS and SP (p<0.001) treatments. However, the highest germination results found in CS were significant over SP (p=1.000). The CS treatment also had the second lowest SE value of 10. 2. The control treatment showed significant difference between CS and AS with *p* values of <0.001 and <0.05, respectively.

## 3. Discussion

In this study, we analyzed the impact of different seed treatment on the germination of 12 plant species native to south Texas, a subtropical semi-arid region. As expected, the there was a wide variability in the viability of the selected species. Seed viability was highest in the forbs, *R. columnifera* and *S. calva* (~79%) while it was lowest in *A. angustissima* (1.25%). Generally, the seed viability of native plant seeds is often lower compared to commercially available agronomic species, where seed viability normally exceeds that of 95%. Seed viability is also dependent on the seed post-harvest management and storage conditions [23], and viability has been found to decline if storage conditions are not appropriate [24]. Thus, reduced seed viability is often expected in commercially available native plants, as a strategy of some native plants to reduce seed herbivory and improve plant fitness.

In addition, seed dormancy is a critical strategy to establishment and plant success, especially for ruderal or stress tolerant plant species. In south Texas and other arid regions, moisture is a limiting factor, and thus one of the major environmental factors in breaking seed dormancy [25]. Our germination results show that seed presoaking is associated with the highest seed germination rates in half of the selected plant species. The water soak, exclusive to the aerated hydroprime treatment likely enhanced the endosperm through imbibition. Hydropriming can also increase the activity of enzymes involved in seed germination [26]. In our study, seed presoaking resulted in 100% germination in *P. bicolor*, a bunchgrass. While the total germination rate in *R. columnifera* seeds treated with seed presoaking was not significantly different than the control at the end of the study period, resulted in germination 6x earlier. Early germination is crucial for ruderal species in arid and semi-arid regions as plants can compete through preemption for limited resources such as soil moisture [27,28]. The early onset of seed germination also prevents the threat of seed predators [29,30], while facilitating the earlier accumulation of ecosystem services such as prevention of soil erosion and pollutant sequestration [4].

Seed scarification—which alters the seed coat and makes water and gases permeable—is also a commonly used pre-sowing method, especially for seeds with hard seed coat. In our study both acid scarification and sand scarification had species-specific impacts. Our results show that acid scarification negligibly improved germination rates in *W. acapulcensis* (2.1% versus 0%) compared to other treatments. [31] reported a high germination rate (77%) in *A. angustissima* seeds treated with acid; however, in our study, the germination rate after acid scarification was not significantly different from control. It should be noted that the type and concentration of acid and time of seed exposure is known to affect germination [32], clearly more detailed experimental design that incorporates these variables is warranted. Similarly, sand scarification, a mechanical scarification technique, was most effective in *D. virgatus* and *A. angustissima,* resulting in highest germination among all treatments. Our results are consistent with that of [33], who demonstrated that mechanical and chemical scarification have been reported to be effective in breaking dormancy in forage legumes with hard seeds. Interestingly, cold stratification treatment which imitates natural winter was only effective in increasing the germination rate of *S. calva* and had no impact on the germination of other plant species. We speculate that this could be due to the fact below freezing temperatures are uncommon in this semi-arid region of south Texas, and thus this pre-sow treatment would have little effect on seed germinability for plants native to this area.

## 4. Materials and Methods

### 4.1. Study Species

We selected 12 common native to south Texas, which included four grasses, four legumes, and four forbs (Table 2). These species represent plants used in habitat restoration projects across the state and are commercially available. For each of these species, we tested seed viability and seed germinability.

### 4.2. Seed Viability 

To examine seed viability, we treated a subset of each species (10 per species) to 4 mL of a 1% 2,3,5 triphenyl tetrazolium chloride (TTC), which stains mitochondrial respiring tissues [46]. The seeds of two hard-coated legume species (*L. texensis* and *A. angustissima*) were imbibed in DI water for 24 h before staining. The treated seeds were placed in a Petri dish and sealed with parafilm (Bemis Company, Inc.; Neenah, WI), and placed in dark at room temperature for 3–4 d, after which seeds were dissected and the total number of stained (viable) seeds of each species was recorded.

### 4.3. Germination Trials 

Ca. 500 seeds of each species were sterilized with sodium hypochlorite following a modified protocol by [47]. The seeds were soaked in dilute bleach (3% sodium hypochlorite solution) for 10 min in an incushaker (Benchmark Scientific, Sayreville, NJ, USA). The seeds were then rinsed with deionized water. From this, ca. 100 seeds of each species were subjected to one of four different pre-sow treatments: sandpaper scarification, acid scarification, cold stratification, aerated hydropriming, as detailed below. Another set of seeds were set aside for a control (no pre-treatment). After treatment, randomly selected seeds of each species were placed separately in a 10 Petri dishes lined with Whatman no.1 filter paper (10 seeds per plate). A volume of 4 mL of DI water was added to each plate, sealed with parafilm, and placed in an environmental chamber with 14:10 light/dark cycle, 27 °C, and 65–70% RH for 10 days [48,49]. Total number of germinated seeds in each treatment was counted after 2 days to determine early germination and at day 10 to determine the total germination rate.

*(a)*  Sandpaper Scarification (SS)

The seeds of the Poaceae family (*P. bicolor*, *C. cucullata*, *C. subdolistachya*, and *B. repens*), which are comparatively smaller in size, were placed between two pieces of 60-coarse sandpaper (#3, St. Paul, MN, USA) and hand scrubbed while the bigger seeds of Asteraceae (*R. columnifera*, *S. calva*, *G. pulchella, W.*) and Fabaceae (*D. virgatus, L. texensis, A.angustissima, D. purpurea*) were shaken in a glass jar lined with 60-coarse sandpaper for one minute before plating.

*(b)*  Acid Scarification (AS)

We soaked 100 seeds of each of the study species in 10% H_2_SO_4_ for 50 min. The seeds were then strained using coffee filters and rinsed with di-water three times before placing seeds into the Petri dish.

*(c)*  Cold Stratification (CS)

A total of 100 seeds of each species were wrapped separately in a damp paper towel. Excess water was removed and paper towels with seeds were placed in plastic cups. The cups were then stored in a freezer (−18 °C) for 60 days.

*(d)*  Seed Presoaking (SP)

Seeds of all study species were separately placed in drawstring bags and tied to a 2.26 kg weight plate. The seeds were then placed in a 0.075 cubic meter (AquaPhoenix Scientific, Hanover, PA, USA) filled with 0.0038 cubic meter of water for 24 h. The seeds in the tank were treated with an aquarium aerator (AquaCulture, Bentonville, AR, USA). After 24 h, the seeds were removed from the bags, and any excess water removed by dabbing with paper towel before being placed in Petri dishes for germination study.

*(e)*  Control

For the untreated control, 100 sterilized seeds of each species were placed in Petri dished separately. As with other treatments the control seeds were treated with 4 mL of DI water.

### 4.4. Data Analysis

The analysis related to the viability was performed using Welch ANOVA followed by the Bonferroni-adjusted pairwise *t*-tests. Two-way ANOVA followed by Tukey’s HSD post hoc comparisons were used to analyze the germination variations between species, treatments and their interactions. Please note that the normality assumption needed for two-way ANOVA was not satisfied. Therefore, we have performed appropriate non-parametric tests including the Kruskal–Wallis test, the Mann–Whitney U test and align rank ANOVA and found similar results which were consistent with the parametric test findings. Since ANOVA is robust against the normality violations [50], we have reported parametric test results in the manuscript. All tests were two-tailed and performed at a significance level of 0.05 using R version 3.6.3 (R Foundation for Statistical Computing, Vienna, Austria).

## 5. Conclusions

An effective seed treatment that improves germination without causing seed mortality can greatly improve the cost effectiveness of native plant restoration projects. Our results shows that the effectiveness of seed treatment varies among different seed species. However, among the four commonly used treatments, aerated hydropriming was the most effective at improving germination for most of the species we tested. Soaking seeds before planting or the use of hydro mulch in seed application can be a potential strategy to maximize germination of native species included in restoration efforts. Future experiments should focus on identifying possible combinations of other seed treatment techniques including plant hormones [51] that could further enhance germination without affecting viability.

## Figures and Tables

**Table 1:** Median germination rate and viability of the selected 12 species.

Species	Mean/Median Germination (*n* = 100) Rate (SD)	Median Viability (%)
*Ratibidia columnifera*	54.8/62 (24.2)	78.7
*Chloris cuculllata*	36.7/40 (20.0)	50.5
*Pappophorum bicolor*	46.4/32.5 (35.7)	75.2
*Simsia calva*	22/21.5 (15.5)	76.3
*Lupinus texensis*	13/13 (4.62)	36.7
*Desmanthus virgatus* var *depressus*	11.8/8 (13.4)	21.4
*Bouteloua repens*	7.38/6.5 (4.99)	49.2
*Acaciella angustissima*	5.08/3 (4.91)	12.3
*Chloris subdolistachya*	4.78/2 (6.52)	24.8
*Dalea purpurea*	2.38/2 (2.07)	59.0
*Gaillardia pulchella*	4.6/0 (9.99)	35.5
*Wedelia acapulcensis* var. *hispida*	0.42/0 (1.2)	36.6

Significant differences in seed viability were also observed among the different species (F(11, 41)=167.58, p<0.001) with a large effect size (η2=0.98;95% CI:0.96, 0.99) (Figure 1). Bonferroni-adjusted pairwise *t*-test reveals significant differences in median viability among 56/66 different pairwise species comparisons. There was significant difference in viability among legume species *D. virgatus* and *D. purpurea (*p<0.001), flowering forb species *R. columnifera* and *Giallardia pulchella* (p<0.001), and *S. calva* and *G. pulchella* (p<0.001), and between grasses *B. bicolor* and *C. subdolistachya* (p<0.001). *R. columnifera*, *C. cuculatta*, *P. bicolor*, and *S. calva* exhibited strong prospects for germination with >70% viability.

**Table 2:** Description of study species including scientific, and common names, plant type, characteristics supporting species selection, seed source, seeding and previously reported germination rates and their citations.

Plant Type	Common Name	Scientific Name	Characteristics	Reference
Grass	Slender Grama	*Bouteloua repens*	Drought tolerant Perennial grass Highly competitive with invasives	[34,35]
	Hooded Windmill Grass	*Chloris cuculllata*	Perennial grass Livestock forage High potential for habitat restoration	[36]
	Pink Pappusgrass	*Pappophorum bicolor*	Perennial grass Livestock forage High potential for rangeland restoration	[37]
	Shortspike Windmill Grass	*Chloris subdolistachya*	Perennial grass Used in roadside and rangeland restoration Highly competitive with invasives	[38]
Legume	Purple Prairie Clover	*Dalea purpurea*	Perennial herbaceous plant Nectar and pollen attract diverse insects Improves soil nutrient status	[39]
	Prairie Acacia	*Acaciella angustissima*	Perennial herbaceous plant Wildlife and livestock forage High potential for habitat restoration, soil reclamation sites	[40]
	Prostrate Bundleflower	*Desmanthus virgatus* var. *depressus*	Perennial herb Forage for cattle and white-tailed deer Seeds are food for bobwhite quail, Rio Grande turkey	[41]
	Blue Bonnet	*Lupinus texensis*	Winter annual flowering plant Attractive to pollinators	[42]
Forb	Indian Blanket	*Gaillardia pulchella*	Annual flowering plant Livestock forage Reseeds easily, easy to maintain Commonly used in roadside plantings	
	Mexican Hat	*Ratibidia columnifera*	Perennial wildflower Young leaves used for livestock grazing The seeds feed birds and small mammals. Nectar and pollen attract diverse insects	[43]
	Bush Sunflower	*Simsia calva*	Simi-woody perennial forb Palatable to sheep, goat, deer, and bird. The border patch butterfly caterpillar feed on the leaves. Nectar plant for insects	[44]
	Orange Zexmenia	*Wedelia acapulcensis* var. *hispida*	Perennial flowering plant Drought tolerant Recommended for landscaping on roadsides and native gardens Browsed by deer, cattle, sheep, goats, bobwhite quail. Nectar plant for butterflies, bees, and other nectar-loving insects	[45]

## Data Availability

All data will be publicly available on dryad.
